# Adult safeguarding managers' understandings of self‐neglect and hoarding

**DOI:** 10.1111/hsc.13841

**Published:** 2022-05-17

**Authors:** Jennifer Owen, John Woolham, Jill Manthorpe, Nicole Steils, Stephen Martineau, Martin Stevens, Michela Tinelli

**Affiliations:** ^1^ NIHR Policy Research Unit in Health and Social Care Workforce The Policy Institute, King's College London London, WC2R 2LS UK; ^2^ Care Policy and Evaluation Centre London School of Economics and Political Science London, WC2A 2AE UK

**Keywords:** adult safeguarding, Care Act, hoarding, older people, self‐neglect, social care

## Abstract

Self‐neglect and hoarding are behaviours that are hard to define, measure and address. They are more prevalent among older people because of bio‐psycho‐social factors, which may be exacerbated by advancing age. This paper aims to further understandings of self‐neglect and hoarding in England's Care Act 2014 context, drawing on a study involving qualitative interviews with local authority adult safeguarding managers who play an important role in determining interventions with individuals who self‐neglect and/or hoard. Online interviews were conducted with adult safeguarding leads and managers from 31 English local authorities in 2021. Interview data were subject to thematic analysis. This paper explores the commonalities and differences in adult safeguarding managers' understandings of the causes and consequences of self‐neglect and/or hoarding among older people, which are likely to have tangible impacts on service provision in their local authority, and influencing of wider changes to policies and procedures. Most participants understood these phenomena as caused by a range of bio‐psycho‐social factors, including chronic physical conditions, bereavement, isolation. A minority took a more clinical or psycho‐medical perspective, focusing on mental ill‐health, or referred to the social construction of norms of cleanliness and tidiness. Whatever their understanding, by the time such behaviours are brought to the attention of safeguarding professionals a crisis response may be all that is offered. The implications of the findings are that other agencies should be encouraged to provide more early help to older people at risk of self‐neglect and/or of developing harmful hoarding behaviours, and that sustained engagement with those affected may help to understand some of the causes of these behaviours to enable effective support or practice interventions.

**What is known about this subject?**

- Definitions of both self‐neglect and hoarding vary and so prevalence estimates vary.
- Causes of self‐neglect and hoarding among older people may be multiple and entrenched.
- Supporting people who self‐neglect or show hoarding behaviours may be professionally and personally challenging.

**What this paper adds**

- Adult safeguarding professionals perceive the causes and consequences of hoarding and self‐neglect as closely related.
- Older people who are at risk from self‐neglect or their hoarding behaviours often come to service attention at crisis point.
- Earlier help and identification of emerging problems from self‐neglect and/or hoarding may help with effective responses.

## 1 INTRODUCTION

Self‐neglect and hoarding are difficult to define, measure and address. In England, as in many other countries, a standard definition for self‐neglect is yet to be agreed. However, ‘hoarding disorder’ was revised from being a diagnostic criteria for obsessive–compulsive disorder (OCD) (Mataix‐Cols et al., 2010) to a new code, first in the Diagnostic and Statistical Manual of Mental Disorders (DSM) code (DSM‐5, American Psychiatric Association, 2013) and endorsed in the 11th revision of the ‘International Classification of Diseases’ (ICD‐11, World Health Organisation, 2021). In the DSM‐5 and ICD‐11, hoarding disorder is defined as a persistent difficulty in discarding possessions, resulting in cluttered living spaces which compromises their intended and safe use. Self‐neglect was written into safeguarding guidance in England under the Care Act 2014, as ‘cover[ing] a wide range of behaviour [including] neglecting to care for one's personal hygiene, health, or surroundings and includes behaviour such as hoarding’ (Anka et al., 2017), so hoarding is seen as both a separate disorder and as a sub‐category of self‐neglect. While giving lawful justification to state intervention in the lives of adults needing care and support who are at risk of harm or neglect, the inclusion of self‐neglect in the Care Act guidance was deemed ‘an afterthought’ that gave rise to some practice uncertainties (Carter, 2016).

Lack of definitional clarity, particularly of self‐neglect, means that prevalence studies are inconsistent and few cover the general population (Mason & Evans, 2020). In England, local authorities (LAs) and the National Health Service (NHS) have not generally collected separate data on self‐neglect and therefore reliable estimates of prevalence are unavailable. One United States' (US) study estimated the prevalence of self‐neglect among people aged 65+ stood between 7.5% and 10.1% (Dong et al., 2012). One study, carried out after the introduction of the DSM‐5 definition, estimates the prevalence of compulsive hoarding/hoarding disorder in adults of all ages in the United Kingdom to be 1.3% (Nordsletten et al., 2013). Concerning the prevalence of hoarding behaviours in older age, Nordsletten et al. (2013, p. 449) further reported ‘that hoarding disorder is more prevalent in older adults’, although Bratiotis et al. (2016, p. 408) cautioned that it may not be that hoarding increases with age but disapproval and ability to cope.

As Day (2020, p. 93) observed, the ‘absence of a universal definition and subjectivity in assessment has been problematic for research and practice’. Indeed, professionals can find self‐neglect and hoarding cases practically, personally and ethically challenging (Braye et al., 2011; Day et al., 2012; Gunstone, 2003).

This present paper aims to further understandings of self‐neglect and hoarding in the Care Act 2014 context, drawing from an exploratory analysis of the views and knowledge of LA adult safeguarding managers who play an important role in managing support and interventions with individuals who are experiencing these problems. Their understandings of the causes and consequences of self‐neglect and hoarding are likely to have a tangible impact on triaging and service provision in their LA, and influence wider changes to policies and procedures. Interviews were undertaken in the first phase of an ongoing study (Social care responses to self‐neglect and hoarding among older people: What works in practice?), which explores the experiences of older people, their relatives and frontline practitioners in the context of hoarding and/or self‐neglect.

## 2 BACKGROUND

Providing successful interventions to help people who are self‐neglecting and/or hoarding is challenging. Most easily identified by their symptoms and consequences, the conditions often encompass personal inability to care for oneself or one's environment in ways that are socially acceptable. Self‐neglect can negatively affect physical and psychological well‐being, mortality, health and social care utilisation (Dong, 2017). Hoarding behaviour can lead to accumulations of treasured possessions that may become unmanageable, unhygienic or unsafe (Bodryzlova et al., 2019).

Under the Care Act 2014 self‐neglect and hoarding were formally recognised within a safeguarding remit in England, therefore requiring responses from LAs and their partners. If an adult appears to have care and support needs and there are concerns about self‐neglect and/or hoarding, regardless of whether they meet the criteria for service provision (Herring, 2016), a Section 42 safeguarding enquiry may be initiated. The referring agency can bring its concern to the lead agency (the LA), through which multi‐agency working is organised, so that fuller assessments and safeguarding planning can take place. In practice, local teams/authorities work differently; clearly defined safeguarding ‘pathways’, which are time or resource constrained (Ash, 2013), do not necessarily well serve people who self‐neglect and/or hoard, and it can be whomever has won the person's trust who acts as a bridge to other interventions (Emmer De Albuquerque Green et al., 2021).

The extent to which people may be choosing to adopt a particular lifestyle (Lauder, 2001, p. 547) presents difficult legal, ethical and practical dilemmas for practitioners. Assessments which attempt to differentiate between ‘an inability and unwillingness to care for oneself, and perceived capacity to understand the consequences of one's actions, become therefore crucial determinants of professional responses’ (ibid.).

According to Braye et al. (2014), the evidence for effective self‐neglect practice points to a combination of negotiated services and imposed interventions based on long‐term relationship‐based work, including assertive outreach and detailed risk and mental capacity assessments, supported by practitioners' legal and ethical literacy. Long‐term involvement is often necessary because people who self‐neglect and/or hoard are otherwise frequently referred repeatedly to adult safeguarding (Rowan et al., 2020). However, this long‐term support can be difficult for managers to justify when, given the deep‐rooted or complex nature of self‐neglect and/or hoarding behaviours, service outcomes seldom restore the person to a pre‐existing or normative state of well‐being and this leads to inconsistent practice.

### 2.1 Theoretical perspectives

Explanatory models of self‐neglect (see Martineau et al., 2021) and/or hoarding (see Steils et al., 2022) focus on different causal factors, interventions and outcomes.

For example, a psycho‐medical paradigm sees self‐neglect as a psychiatric diagnosis (Lauder et al., 2005b), the product of an underlying mental health problems or pathological personality. The recognition of hoarding as a recognised disorder, often, but not always, associated with other disorders such as obsessive compulsive disorder (OCD) (whereby an individual experiences intrusive thoughts and compulsive behaviours around acquiring possessions and difficulty discarding them) (Wheaton, 2016), also reflects a psycho‐medical approach.

Genetic models consider hereditary factors in hoarding behaviour (Iervolino et al., 2009). Neurological explanations consider neural impairment and neurodegenerative conditions as a cause for self‐neglect and hoarding (Hombali et al., 2019; Thew & Salkovskis, 2016; Wheaton, 2016). A social constructionist view argues that self‐neglect should be understood in relation to cultural and historical norms of hygiene and cleanliness (see also Cox, 2011; Lauder, 2001). This paradigm views self‐neglect not as an objective phenomenon, but produced by social, cultural and professional judgement set against mainstream values. Similar arguments are made by Shaeffer (2017) in the context of hoarding. Tolerance of eccentricity (Lauder et al., 2005a) and accumulated possessions (McDermott, 2008) also impacts upon how self‐neglect and hoarding are positioned in public discourse. Consequently, this approach emphasises the importance of professional judgement and negotiated meanings (Lauder, 2001).

The bio‐psycho‐social model considers the interrelation of internal and external factors and their association with, if not causation of, self‐neglect (see Iris et al., 2010). Cognitive behavioural approaches (Taylor & Jang, 2011) were initially developed by Frost and Hartl (1996) for hoarding, later refined by Steketee and Frost (2003, 2007). These have found wider acceptance in practice. These models propose that self‐neglect and hoarding arise from patterns of thoughts, beliefs and behaviours, which therefore suggests intervening in these beliefs and thoughts (Wheaton, 2016).

Life changing experiences and traumatic life events—such as loss of a loved one, illness or abuse and neglect—were considered as causal in hoarding behaviour in several models and evidence of a correlation has been found in some studies but not all (Dozier & Ayers, 2017; Hombali et al., 2019).

Practical, as well as theoretical, understandings of the causes and consequences of self‐neglect and hoarding by adult safeguarding managers are the focus of this paper—and in the next section we outline the research methods employed to uncover them.

## 3 METHODS

The interview guide was developed in consultation with the study advisory group, including older people with direct experiences of hoarding, and safeguarding and hoarding behaviour experts. Interviews were undertaken in 31 LAs with the respective 31 adult safeguarding leads. In 10 LAs, at the request of the safeguarding leads, 13 additional managers were interviewed in joint interviews. This has resulted in the provision of sometimes different and extended information on the application of policies, but on the whole the content and tone of discussions with more than one participant did not differ much from those with single participants. To ensure the sample was geographically dispersed, we invited three LAs from each of the nine English regions. Before each interview (conducted November–December 2020), participants received information sheets and consent forms with a summary of the topics to be discussed. Participants' demographic details are presented in Table 1. Ethical approvals for this phase of the study were obtained from (HRA ref: 21/WM/0109).

**TABLE 1:** Demographic profile of adult safeguarding managers and others interviewed (n = 44)^a^

Total number of participants	44
Gender	Male 10 (28%) Female 26 (72%) Not reported 8
Mean age	48.6 years (n = 26)
Ethnic background	White British 31 (78%) White other 8 (20%) Asian British 1 (3%) Not reported 4

a Some interviews included other managers at the request of the adult safeguarding lead.

Semi‐structured interviews were used as a means of gathering perceptions and capturing a body of practice knowledge. The topic guide was closely followed to produce comparable data across the sample. Due to the COVID‐19 pandemic, interviews were conducted by two researchers (JW and SM) via video conferencing software and audio recorded with consent. On average, interviews lasted about 75 minutes (ranging from 45 to 120). All audio recordings were transcribed and anonymised.

The two interview questions on which this paper specifically draws are:

1. What are the most common factors that lead to self‐neglect and/or hoarding among older people?
2. Based on your experience, what would you say are the most common consequences of self‐neglect and/or hoarding among the older population?

A further question covering the impact of Covid‐19 was asked and these findings are reported elsewhere (Manthorpe et al., 2022).

Thematic analysis, using an analytic induction methodology of the data, was used to distil common topics and understanding. NVivo qualitative data analysis software was used to store, manage and analyse the data. Analysis was carried out collaboratively to review emerging themes as they were identified by the research team that included gerontologists with social care and safeguarding expertise. While attempts were made to code responses related to hoarding and self‐neglect separately, most participants did not make such distinctions, and indeed commented on their inter‐relationships, as described below.

## 4 FINDINGS

This section outlines participants' understanding of the causes of self‐neglect and/or hoarding. Where possible we note if the data relate to self‐neglect, hoarding or both, but often participants did not provide this clarification, perhaps, as discussed later, indicating their understandings of each. Quotes from participants refer to case examples and it is worth noting that the severity of those they chose to recount are at the higher end of what is typical of most people who self‐neglect and/or hoard and may say something about participants' understandings and the emotionality of the work.

### 4.1 Getting to the problems' roots

Complex and interrelated causes were said to make self‐neglect and/or hoarding difficult to define and provide successful interventions to improve people's well‐being. Several participants reported difficulty understanding the causes of self‐neglect and/or hoarding since cases presented late:

Sometimes it's been an issue throughout a person's life, but social care perhaps don't come into contact until it hits a crisis or they have physical needs. So often it's been something we've not been aware of, by the time we are aware of it, it tends to be at a crisis point, I think. (LA11)

Most considered there were multiple intertwined factors or that behaviours were deep‐rooted. Cases were therefore unique:

Each case, I think, is unique to the person, [and] without being too simplistic here, the opportunity for effective intervention, I believe, needs to identify, as you said, what the trigger is, what the cause is, what's driving the behaviour and, until we have a concerted attempt to address that, we will never be able really to support the person. (LA20)

### 4.2 Construction of self‐neglect and hoarding ‘cases’

Differences between the norms of self‐care and household maintenance of older people and of families and professionals working with them were considered important to the construction of self‐neglect and/or hoarding:

I think people's standards vary, some people live in a very different way, and what is considered normal and appropriate standard of living for one person is very, very different for somebody else. […] [B]ut obviously if somebody goes in that has a very different idea of what appropriate standard of living is, then they would see that as self‐neglect. (LA02)

Having prior knowledge of what is ‘normal’ for an individual was thought to be important to help identify any change worsening their circumstances. Identifying hoarding seemed more difficult because, outside their home, behaviours could be hidden, and because not everyone presenting with hoarding behaviours also self‐neglects.

#### 4.2.1 Chronic conditions, triggering events and beliefs

The three most commonly mentioned types of causes were chronic conditions, triggering events and beliefs.

#### 4.2.2 Chronic conditions and causal factors

Reflecting a psycho‐medical approach, mental ill‐health and sometimes physical health problems were cited as primary causes of self‐neglect and/or hoarding. Conditions such as disability, stroke and diabetes were mentioned by a few participants in relation to reduced mobility, both in terms of bringing items closer to hand and difficulty maintaining cleanliness and hygiene.

In contrast, several mental health conditions were cited by participants, most commonly depression, post‐traumatic stress disorder (PTSD) and obsessive compulsive disorder (OCD), as being linked to hoarding and/or self‐neglect. One considered that the recent DSM‐5/ICD‐11 recognition of hoarding as a mental disorder was helpful in explaining and framing responses to hoarding behaviour. Another participant thought that entrenched psychological factors leading to hoarding and/or self‐neglect were harder to address.

A lack of formal diagnosis of mental ill‐health over the years was mentioned several times as impacting on older people. This related both to improvements in clinical and popular understandings of mental health over time and the complexity of cases leading to non‐ or mis‐diagnosis. There was also reference to those who had been ‘kept in the family’ whose needs only emerged when relatives died.

Other participants linked mental ill‐health to other factors, including substance abuse or trauma, or suggested these were the underlying cause:

We've got lots of cases coming through where, because of their deteriorated mental health, often because of substance misuse and the challenges around their substance misuse, that they start to deteriorate, their ability to self‐care, their ability to self‐nurture, maintain sufficient nutrition. (LA23)

#### 4.2.3 Triggering events

In addition to long‐standing conditions, triggering events were often mentioned as prompting self‐neglect and/or hoarding behaviours. Connections were most commonly drawn with hoarding but were not restricted to this.

Loss, of anything—partner, job or good health—but particularly bereavement, was identified as a type of traumatic reaction that could lead to the loss of identity or co‐dependent relationship, and therefore the beginning or exacerbation of self‐neglect and/or hoarding behaviours:

It can also be a series of smaller losses that then… it's just one loss too many, and that could be job, it could be health, it could be the loss of a relationship that's not necessarily a bereavement, but it all seems to be linked to loss in our experience. (LA19)

Other triggering instances from childhood, such as abuse, poverty or wartime rationing, were also mentioned.

The trauma of an event was described as having a profound impact on motivation for some, meaning people would ‘give up’ and either start self‐neglect and/or hoarding behaviours or quickly deteriorate:

(Person) had a physical disability, they'd always been quite an active individual and they were given medical advice that said, this is basically it, there is nothing more we can do for you; your health physically is going to decline very, very rapidly and that was the point they sort of gave up, I guess, and that's when the self‐neglecting started because they couldn't really see a life beyond that. (LA28)

#### 4.2.4 Beliefs

The final type of cause for self‐neglect and/or hoarding identified centred around people's beliefs, in line with a cognitive behavioural explanation of these behaviours. Such beliefs were said to originate from family norms, a desire to withdraw from society, or preferences for alternative medicines or healing. Such beliefs could become entrenched:

I would suggest it's learned behaviour, particularly with regard to the one lady that I mentioned before, she was absolutely adamant that that was all fine because that's how I grew up… she said to me once, I was a baby and crawled around in all this and nothing happened to me, so why would I need to change anything, and she was probably nearing 70 at that point. […] Just always lived that way. (LA24)

Beliefs that professionals would make negative judgements and interfere were thought to lead to people secluding themselves:

They isolate themselves to safeguard them psychologically from intervention from the state, or other people who they perceive may wish to take over control, and the things that they can control is getting stuff into their property and this is how they are managing life. (LA14)

One participant observed that beliefs in the power of alternative medicines or religion led to avoidance of medical assistance, risking self‐neglect and/or hoarding:

We had a safeguarding referral through for a lady who had always used homeopathic medicine and never accepted health services, […] but she had now developed, with age and she was also a hoarder, there was hygiene issues within the home, so her health had progressed to be quite bad, and our health service colleagues were very worried […]. And another lady who thought that God would intervene and had […] developed extreme religious beliefs that she directly related… if I wait long enough, God is going to make this better. (LA24)

### 4.3 Exacerbating factors

In addition to causes, participants also identified factors which exacerbated people's problems with self‐neglect and/or hoarding. The most prominent were age‐related impairment, disability or frailty, making situations increasingly untenable. Also, alcohol and other substance abuse could underlie behaviours, both exacerbating and masking the severity of self‐neglect problems:

They are people who very often will have a whisky before they go to bed, and the whisky becomes two whiskies, and the next thing they're asleep in the chair, or they've just sat in that chair and finished the whisky or finished the wine, they're developing pressure sores because they've been sat in the same place, or their mobility's deteriorating, so they're drinking to manage that, and then the impact on that is deterioration of skin, so pressure areas and things. (LA15)

Deliberate self‐isolation could increase hoarding severity particularly, which again could increase with age as significant relationships were lost:

People don't let people into their environment as much as possible and it leads them to hoard even more because there's nobody asking questions or having a conversation about their belongings that may be causing some problems. (LA14)

### 4.4 Differences between self‐neglect and hoarding causes

Most participants did not differentiate between the causes of self‐neglect and hoarding because they believed ‘the presentation can be very similar’ (LA14). Those who identified differences pointed to the linking of self‐neglect and hoarding in definitions as making distinctions difficult:

Sometimes perhaps in our discourse they have been linked together where that is not always as helpful. (LA20).

Views of the possibility of having one condition and not the other, when the two were so closely related, also differed between participants. Although most thought that one could self‐neglect without hoarding, the contrary situation was only reported by one manager:

[Some] people are very able to self‐care, so they're not actually neglecting themselves, they're just living in a very hoarded and cluttered environment. (LA10)

### 4.5 Consequences

From the ways participants spoke about cases of self‐neglect and/or hoarding it seemed that causes and consequences were often considered inextricably linked where initial causes were exacerbated by the self‐neglect or hoarding (with the exception of fire risk). For example, stigma based on outward appearance and eccentric habits could further reinforce isolation:

I think sometimes the stigmatisation that can go with self‐neglect and hoarding because of how someone outwardly appears to others can actually result in further isolation and further marginalisation, so there's a compounding of the problem from a social point of view, which could actually then create a vicious cycle of perpetual self‐neglect. (LA22)

Another aspect of this interlinking was that the symptoms of self‐neglect and hoarding often resulted from the person's response to the factors causing the behaviour, which could be exacerbated by the self‐neglect and hoarding. For example, LA14 observed that, whatever the reason(s), the consequences of self‐neglect and hoarding could start a downward spiral.

A referral to safeguarding services arising from a crisis was also thought to often lead to assessments focusing on consequences of self‐neglect and/or hoarding behaviour rather than underlying causes because of the necessity to respond to immediate risks and problems.

#### 4.5.1 Serious health implications

Several participants mentioned serious health implications of self‐neglect and/or hoarding, often linked to common age‐related challenges such as poor mobility. The most cited physical health consequences were falls, malnourishment, amputations and ulcers, as the adult safeguarding manager in LA23 recalled:

One example is people refusing to take diabetes medication, for example, so they're neglecting their need to have essential medication, and that result in a deterioration in the physical health … they end up getting gangrene and we've had cases where they've ended up having amputations, really awful. (LA23)

A lack of personal care and refusal of medical treatment could mean pressures ulcers developed. In these rare instances of severe deterioration in health following self‐neglect and/or hoarding the consequences could be hospital admission or death.

#### 4.5.2 Social isolation

Isolation was described both as a cause of self‐neglect and hoarding behaviours, but also a consequence of feelings of shame and embarrassment, and acts of stigmatisation and marginalisation by others:

They're so embarrassed about the state of the house that they don't let people over the doorstep and create all sorts of pretexts and excuses that often result in families being pushed away from them. (LA16)

Experiences of isolation and loneliness were also linked to poor overall well‐being and increased mental health problems.

#### 4.5.3 Poor living conditions

Home conditions were mentioned by several participants, who described environmental risks which could lead to infections, but also general disrepair:

Hoarding can result in very insanitary conditions, particularly if people are hoarding faeces or urine, hoarding old food, obviously all of those things create a massive risk of health problems, infection and of course infestation by other types of animals like rats and cockroaches and so forth. (LA22)

Several participants mentioned cases where people lacked basic utilities, including running water, sanitation, heating and cooking facilities. This may have come about from not allowing people into their homes, or services being unable to access them, and had obvious consequences for hygiene, comfort and nutrition:

We've had a few cases […] where it's not so much hoarding, it's people living without the everyday things that you'd expect them to be able to have. So, there was one chap who the living room didn't look too bad and nobody went further than the living room, but actually he didn't have very basic things, like a working toilet and stuff like that. (LA26)

#### 4.5.4 Fire risk

Risk of fire featured as one of the main consequences by most participants, particularly related to hoarding. Links were made between fire risk and poor amenities, cluttered spaces and smoking:

We've had a couple of fires in [LA15], where people tragically have died as a result of smoking predominantly, in environments that were either cluttered or weren't appropriate for them to reside in. (LA15)

Fire risks could be compounded by hoarded material blocking escape routes and access for emergency services.

#### 4.5.5 Wider community risks

Several participants referred to the wider environmental impact of self‐neglect and hoarding. Sometimes these prompted concern and complaints leading to police involvement, legal action and anti‐social behaviour:

Depending on the extent of it, […] it can also attract anti‐social behaviour, particularly when hoards and evidence of self‐neglect on the environment tips out into the external bits of the property. […] We also got the impact on adjoining neighbours and the community itself, particularly if you're seeing environmental health risks, like vermin being attracted to properties. (LA29)

#### 4.5.6 Loss of autonomy or home

There was some recognition that self‐neglect or hoarding could diminish control or autonomy, linked to hospital admission, removal from or loss of the home. Health deterioration could make return unsafe:

Consequences are enforcement notices; consequences are lots of serious council meetings. […] sometimes cases go to court, sometimes there are notices put on the property, sometimes there are charges [on the] property and sometimes the individual is left with no other option but to sell their property and move into another type of accommodation. (LA14)

Linked to this was an acknowledgement that statutory interventions risked further distress and isolation.

## 5 DISCUSSION AND IMPLICATIONS

Our findings convey a rich picture of cases of self‐neglect and hoarding coming to the attention of adult safeguarding professionals together with their understanding of both causes and consequences. A bio‐psycho‐social model of hoarding and self‐neglect (see Iris et al., 2010) considering the interrelationship of personal, social and environmental causes and consequences appeared dominant. However, some participants also emphasised psycho‐medical factors, such as mental ill‐health, and the importance of behavioural norms and social construction of self‐neglect and hoarding. Their understanding is important as participants are senior, experienced professionals, whose actions can prompt the well‐organised and long‐term multi‐agency response to such problems. How they understand and respond may have the potential to influence decisions at all levels as well as system‐wide procedures and actions.

The paper has limitations in being confined to LA adult safeguarding managers, so missing the perceptions and experiences of frontline colleagues, professionals in other agencies, and the perspectives of older people and their families who may have more recent direct experience of self‐neglect and/or hoarding. However, this was a large and varied interview sample from different English regions meaning that the transferability (Sale & Brazil, 2004) of findings is likely to be high. Our qualitative approach explored understandings and participants often cited case examples to evidence their expertise and experiences. Many of these seemed to be at a severe level, perhaps indicative of their memorability rather than being typical examples. In discussing the data reported above, three overarching themes seemed prominent.

### 5.1 Cases come to safeguarding at crisis point

First, cases of self‐neglect and/or hoarding come to safeguarding's attention at ‘crisis’ or ‘end stage’. Participants provided several reasons for this, some directly related to the triggers or presentations of self‐neglect or hoarding, for example, social withdrawal and healthcare refusal, or the loss of someone supportive. Most acknowledged that self‐neglect and/or hoarding rarely arose suddenly. This points to the need for more work upstream by safeguarding and other community services (or a recalibration of safeguarding thresholds), including knowing what is ‘normal’ for people so that any changes can be assessed more precisely and be acted on sooner, while respecting the older person's wishes, culture and family history. Furthermore, while strongly promoted by the Care Act 2014, resource pressures have side‐lined preventive work (Naughton‐Doe et al., 2020).

### 5.2 Consequences disguise causes

Second, since there is no ‘perpetrator’ (unlike other kinds of abuse or neglect) establishing the causes of self‐neglect and/or hoarding is difficult. The causes, symptoms and consequences of self‐neglect and/or hoarding were deemed hard to disentangle, and while the consequences, as the tangible or visible side of the disorders were easier to recognise, they could mask underlying causes. With more entrenched behaviour, these causes may be particularly difficult to unearth especially if an individual is themself unaware of the triggers (Dozier & Ayers, 2017; Hombali et al., 2019). In a US study of narratives of self‐neglect (Lien et al., 2016), older people revealed isolated traumatic personal experiences—psychologically traumatic loss, separation or abandonment, and violent victimisation, physical trauma or sexual abuse—as possible contributors to self‐neglect. Lien et al. called on professionals to be more alert to such hidden causes. In the present study, it was reportedly difficult for adult safeguarding professionals to address causes, given the late stage at which cases come to their attention. By enabling adult safeguarding managers to be more aware of the potentially complicated origins of self‐neglect and/or hoarding, how they may be conceptualised, and potential early and sustained consequences—through detailed training and guidance—they may be able to encourage more detailed or fuller assessments that are informed by the building of a relationship with the older person concerned. The economic burden of hoarding on health and social services, housing providers, and fire and rescue services is significant, both for immediate risk reduction and sustained interventions, although more research is needed to understand this more fully (Neave et al., 2017). It is known that people with hoarding behaviours are more likely to report a broad range of chronic and severe medical concerns and, as a result, have a fivefold higher rate of mental health service utilisation (Tolin et al., 2008).

### 5.3 Working with severe consequences

Finally, the severity of some outcomes—including amputations, and death from fires or life‐threatening conditions—was mentioned by participants. These accounts may have featured because participants might have believed the researcher wanted to hear about serious harms but more likely could reflect their personal and professional concerns. When talking about life‐threatening conditions and environmental risks, participants conveyed their worries about affected individuals. Regarding practitioners, our data reinforce others' findings that those who work with individuals who self‐neglect or hoard struggle emotionally ‘with extremely difficult and incomprehensible experiences and situations, which evoke a wide range of emotions’ (Band‐Winterstein, 2018, p. 976). This, in turn, lends credence to the argument that practitioners should receive both good supervision and ongoing support in this area of practice where some individuals may be ‘hard to engage, resistant and sometimes hostile’ (Preston‐Shoot, 2019).

## 6 CONCLUSION

There are many assertions that coordinated, multi‐disciplinary, person‐centred interventions are successful in supporting and improving the well‐being of older people who self‐neglect and/or hoard (Martineau et al., 2021; Steils et al., 2022). However, while specialist treatment can reduce symptoms and risks associated with hoarding, these reductions are generally modest (14–40% symptom reduction) and many participants remain in the clinical range of hoarding disorder after clinical treatment (Thompson et al., 2017). So, in reality, ideas of ‘best practice’ when supporting people who self‐neglect and/or hoard derive more from accumulated practice wisdom than formally evaluated interventions (ibid.). What is missing from this evidence is the importance of thorough understandings of the conditions.

This paper analysed the perspectives of adult safeguarding leads and managers about the complex phenomena of self‐neglect and hoarding. Participants often did not differentiate between self‐neglect and hoarding in discussion, perhaps indicating their level of understanding and the combined definition in guidance to the Care Act 2014, as well as a lack of universal definition of self‐neglect, as can be noted in many LA guidance documents on self‐neglect. There was broad agreement about the causes originating from a combination of poor mental health, trauma, beliefs and social factors, as well as a recognition of the importance of potential conflicts between professional and family/individual norms. Such framing shows a preference for a bio‐psycho‐social model of understanding, although the importance of health factors and social construction was also evident, suggesting that these perspectives are not mutually exclusive. Only a handful of participants were ‘outliers’ to this consensus and pointed solely towards medical, namely physical, reasons for the behaviours. However, no matter their understanding of the causes, all participants conveyed a clear understanding of the range and potential severity of the consequences of self‐neglect and/or hoarding on older people's physical and mental health, relationships, and living conditions – which extend beyond the individual to their wider networks.

Better understanding the range of causes of self‐neglect and/or hoarding by adult safeguarding managers (as well as frontline colleagues) may assist effective assessment, engagement, risk management and intervention, and, while probably not ‘fixing’ the problem, aid in the achievement of better outcomes or prevention of severe consequences. This is especially pertinent for older people who can have added complications or risks of health decline. However, current systems mean that cases of self‐neglect and/or hoarding often become known to adult safeguarding teams at ‘crisis’ point, so early reporting and forms of help need to be given greater priority by social care and third‐sector organisations, as well as NHS screening and monitoring, reviews and post‐diagnostic support and carer support.

## AUTHORS' CONTRIBUTIONS

JW and SM conducted the interviews and data were analysed by JO, JM, NS, MS and SM. JO drafted the initial manuscript, with input from the rest of the team including MT. The article was revised by the team. NS and JM lead the overall study.

## CONFLICT OF INTEREST

The authors declare none.

## Funding information

This work was supported by the NIHR School for Social Care Research (102645/CM/KCLJM‐P208); the views expressed in this article should not necessarily be interpreted as that of the NIHR or the Department of Health and Social Care.

## ETHICS

Ethical approval for this project was given by the Health Research Authority, West Midlands Coventry & Warwickshire Research Ethics Committee Ref: 21/WM/0109.

## Data Availability

Due to the nature of this research, participants of this study did not agree for their data to be shared publicly, so supporting data are not available.
